# Glucagonlike Peptide-1 Receptor Imaging in Individuals with Type 2 Diabetes

**DOI:** 10.2967/jnumed.121.262506

**Published:** 2022-05

**Authors:** Olof Eriksson, Irina Velikyan, Torsten Haack, Martin Bossart, Iina Laitinen, Philip J. Larsen, Jan Erik Berglund, Gunnar Antoni, Lars Johansson, Stefan Pierrou, Joachim Tillner, Michael Wagner

**Affiliations:** 1 Antaros Medical AB, Uppsala, Sweden;; 2 Science for Life Laboratory, Department of Medicinal Chemistry, Uppsala University, Uppsala, Sweden;; 3 Department of Medicinal Chemistry, Uppsala University, Uppsala, Sweden;; 4 Akademiska Sjukhuset, Uppsala, Sweden;; 5 R&D Research Platform, Integrated Drug Discovery, Sanofi, Frankfurt, Germany;; 6 Global Imaging, Sanofi, Frankfurt, Germany;; 7 Diabetes Research, Sanofi, Frankfurt, Germany;; 8 Clinical Trial Consultants AB, Uppsala, Sweden; and; 9 Translational Medicine, Sanofi, Frankfurt, Germany

**Keywords:** GLP1R, PET, exendin, type 2 diabetes, β-cell mass

## Abstract

The glucagonlike peptide-1 receptor (GLP1R) is a gut hormone receptor, intricately linked to regulation of blood glucose homeostasis via several mechanisms. It is an established and emergent drug target in metabolic disease. The PET radioligand ^68^Ga-DO3A-VS-exendin4 (^68^Ga-exendin4) has the potential to enable longitudinal studies of GLP1R in the human pancreas. **Methods:** ^68^Ga-exendin4 PET/CT examinations were performed on overweight-to-obese individuals with type 2 diabetes (*n* = 13) as part of a larger target engagement study (NCT03350191). A scanning protocol was developed to optimize reproducibility (target amount of 0.5 MBq/kg [corresponding to peptide amount of <0.2 µg/kg], blood sampling, and tracer stability assessment). The pancreas and abdominal organs were segmented, and binding was correlated with clinical parameters. **Results:** Uptake of ^68^Ga-exendin4 in the pancreas, but not in other abdominal tissues, was high but variable between individuals. There was no evidence of self-blocking of GLP1R by the tracer in this protocol, despite the high potency of exendin4. The results showed that a full dynamic scan can be simplified to a short static scan, potentially increasing throughput and reducing patient discomfort. The ^68^Ga-exendin4 concentration in the pancreas (i.e., GLP1R density) correlated inversely with the age of the individual and tended to correlate positively with body mass index. However, the total GLP1R content in the pancreas did not. **Conclusion:** In summary, we present an optimized and simplified ^68^Ga-exendin4 scanning protocol to enable reproducible imaging of GLP1R in the pancreas. ^68^Ga-exendin4 PET may enable quantification of longitudinal changes in pancreatic GLP1R during the development of type 2 diabetes, as well as target engagement studies of novel glucagonlike peptide-1 agonists.

## Visual Abstract

**Figure:**
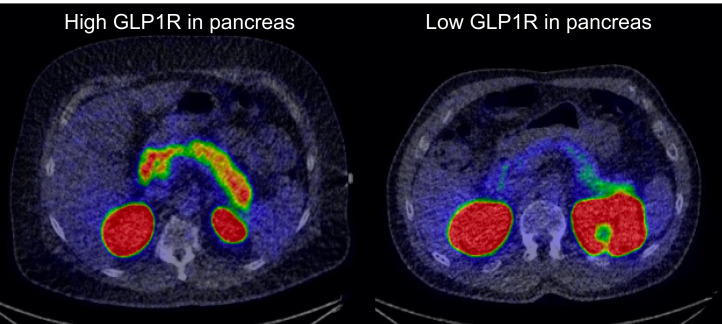

The glucagonlike peptide-1 (GLP1) receptor (GLP1R) is a gut hormone receptor intricately linked to regulation of blood glucose homeostasis via several mechanisms such as insulin secretion, gastric emptying, and control of food intake (*1*). Endogenous GLP1 peptide is released from the intestinal L cells in response to nutrient intake. Synthetic GLP1 agonists are already approved for treatment of such conditions as type 2 diabetes (T2D), and further insight into their mechanism of action is still of the utmost interest. Several recent studies have demonstrated beneficial effects of GLP1 agonists on hemoglobin A1c, cardiac function, and survival (*2*,*3*).

Exendin4 is a synthetic peptide that binds to GLP1R with nanomolar affinity and high specificity (*4*). The development of ^68^Ga-radiolabeled analogs of exendin4 (*5**,**6*) has recently enabled noninvasive PET imaging in humans, primarily for the diagnosis and localization of insulinomas (*7*,*8*), as well as expression of GLP1R in the pancreas.

The possibility of longitudinal imaging to quantify GLP1R density in the pancreas is of interest both in the assessment of drug mechanisms of action (e.g., in the context of unimolecular dual and trigonal agonists) (*9*,*10*) and because of the potential association between GLP1R expression and the remaining β-cell mass (*11*). Here, we used ^68^Ga-DO3A-VS-exendin4 (^68^Ga-exendin4) for PET imaging of GLP1R in the human pancreas. The current study was performed as part of a clinical trial investigating the target engagement of a novel GLP1/glucagon receptor dual agonist, SAR425899, in individuals with T2D (clinicaltrials.gov identifier NCT03350191) (*12*).

Here, we demonstrate for the first time—to our knowledge—the noninvasive quantification of GLP1R density in the pancreas of individuals with T2D by PET imaging. We furthermore outline a protocol for reproducible, longitudinal, and accurate ^68^Ga-exendin4 PET/CT scanning and explore the origin of the variability of pancreatic GLP1R density this patient population.

## MATERIALS AND METHODS

### Nonhuman Primate (NHP) PET Study

A ^68^Ga-exendin4 PET/CT imaging study on healthy cynomolgus NHPs was in part reported and described in detail previously (*5*). Briefly, NHPs were scanned by ^68^Ga-exendin4 PET using a dose escalation study design in which several examinations were performed, several hours apart, over the course of one or several experimental days. Each subsequent scan entailed a higher peptide mass and radioactive dose to minimize influence from the preceding scan (Table 1). The resulting dataset enabled analysis of the self-blocking effect of the increasing amounts of coinjected unlabeled exendin4 precursor peptide.

**TABLE 1.** Overview of Dose Escalation of ^68^Ga-Exendin4 and Coinjected DO3A-VS-Exendin4 in NHPs

		NHP				
Scan	Unit of measurement	1	2	3	4	5
	kg (body weight)	5.6	5.5	7.4	9.0	6.0
1	µg/kg	2	0.05	0.15	0.05	0.0025
	MBq	11.1	2.0	4.6	2.3	0.2
2	µg/kg	—	1	20	1	0.5
	MBq	—	6.5	11.1	6.7	6.9
3	µg/kg	—	10	—	3	15
	MBq	—	5.2	—	4.9	18.7

NHPs 2 and 5 were same individual scanned twice, 6 mo apart.

Previously, only the simplified SUV analysis of 3 of the NHPs (8 scans) was reported (*5*). In preparation for the design of the clinical study, to better understand the peptide doses at which self-blocking starts to occur, we reanalyzed the dynamic NHP data from all 12 scans by graphical analysis using an image-derived blood plasma input.

Pancreas and other tissues of interest (such as spleen as a negative reference tissue) were delineated using PMOD, version 4.0 (PMOD Technologies). The PET signal for each tissue and time frame was corrected for the injected amount, and the image-derived input function was extracted by segmenting voxels fully within the lumen of the descending aorta as identified on early PET frames and coregistered CT projections. The aorta signal was further corrected for the plasma partition.

The volume of distribution (V_t_) of ^68^Ga-exendin4 in the pancreas was estimated by graphical analysis according to Logan by applying the image-derived input function using the PMOD PKIN module (*13*). Patlak graphical analysis was also attempted (*14*), assuming irreversible uptake of ^68^Ga-exendin4 in tissue due to receptor agonism and internalization, but this model failed in some individuals and is not reported.

The pancreatic V_t_ for each scan was plotted against the amount of coinjected unlabeled precursor peptide to explore the self-blocking at different doses.

Furthermore, the pancreatic V_t_ was plotted against the static uptake values at different time points, to determine whether static SUVs could replace full dynamic PET scans with invasive plasma sampling.

### Clinical Study Design

These ^68^Ga-exendin4 PET/CT examinations were acquired as part of a phase Ib single-center, open-label study assessing the glucagon receptor and GLP1R occupancy of dual-agonist SAR425899 in overweight-to-obese T2D patients (clinicaltrials.gov identifier NCT03350191). Individuals with T2D (*n* = 13) were recruited and underwent PET/CT scanning for GLP1R availability in the pancreas (^68^Ga-exendin4) and glucagon receptor availability in the liver (^68^Ga-DO3A-VS-Tuna-2) at baseline. Participants were then treated with up to 0.12 or 0.2 mg of SAR425899 daily from 3 wk, followed by on-drug scanning with ^68^Ga-exendin4 and ^68^Ga-DO3A-VS-Tuna-2. Six participants completed the full NTC03350191 occupancy study, and the results from those individuals, especially the occupancy of SAR425899 as evaluated by PET, were previously reported (*12*). This study reports the results of all 13 baseline ^68^Ga-exendin4 PET scans and the details of the procedures. The baseline examination of the 13 ^68^Ga-DO3A-VS-Tuna-2 examinations was similarly reported independently (*15*).

### Patient Population

Overweight-to-obese individuals diagnosed with T2D (*n* = 13) were recruited to the study. The participants had a median age of 69 y (range, 50–76 y) and a mean body mass index (BMI) of 31.2 ± 3.0 kg/m^2^. Of the 13 participants, 12 were men and 1 was a woman. Patients were not allowed to be on any antidiabetic medication during the study except for stable metformin or sulfonylurea treatment.

No control group with nondiabetic individuals was included, since the full study was aimed at assessing drug efficacy and occupancy and thus included 3 wk of drug treatment with SAR425899.

All study participants provided written informed consent. Study protocols were approved by national health authorities and an independent ethics committee, and the trial was performed in accordance with the guidelines established by the Declaration of Helsinki and the International Conference on Harmonization–Good Clinical Practice.

### PET/CT Examinations

Good-manufacturing-practice DO3A-exendin4 was provided by Sanofi. Good-manufacturing-practice–quality ^68^Ga-exendin4 was produced on an automated synthesizer (Modular Lab Pharm Tracer; Eckert and Ziegler) as developed and reported previously (*12*,*16*). The radiochemical purity was over 90%, with no unknown single impurity of over 5%.

The PET assessments were performed 3 h after a standardized meal followed by fasting, to minimize the variability in GLP1 levels at the time of scanning. The individuals were examined over the abdomen with a Discovery MI PET/CT scanner (20-cm field of view; GE Healthcare). Low-dose CT was performed for attenuation correction and anatomic coregistration of PET images. The dose of the CT scan was limited by dosimetry considerations, given that each individual underwent up to 4 PET/CT examinations over the entire clinical study.

A 0.5 MBq/kg target dose of ^68^Ga-exendin4 (0.46 ± 0.03 MBq/kg, corresponding to 0.14 ± 0.04 µg of peptide per kilogram) was administered intravenously as a bolus. The amount of administered ^68^Ga-exendin4 was based primarily on limiting the associated dose of DO3A-VS-exendin4 precursor peptide to below 0.2 µg/kg. This limit was imposed to minimize any self-blocking, and the cutoff was determined from the NHP dose escalation studies.

Dynamic PET was initiated at administration and continued for 60 min. Blood sampling for glucose was performed before and during the scan as a safety precaution, as exendin4 can stimulate insulin secretion at pharmacologic doses. For 3 individuals, arterial sampling was performed at 5, 30, and 60 min after ^68^Ga-exendin4 administration to measure the radioactivity in whole blood and plasma and to determine the metabolic stability of the tracer (the methods are shown in the supplemental materials, available at http://jnm.snmjournals.org) (*17*).

PET images were reconstructed using an iterative VUE Point FX-S algorithm (GE Healthcare) (3 iterations, 3 subsets, 256 × 256 matrix, 3-mm *z*-axis postprocessing filter) with all relevant corrections performed (30 frames in total: 12 × 10 s, 6 × 30 s, 5 × 120 s, 5 × 300 s, 2 × 600 s).

### PET Image Analysis

Abdominal tissues of interest, including pancreas (target tissue), aorta (input signal), kidney (excretion), and spleen and erector spinae muscle (negative reference tissues) were manually segmented on coregistered PET/CT images using Carimas software, version 2.9 (Turku PET Center). The aorta was delineated by segmenting single voxels fully within the lumen of the descending aorta. The arterial plasma image-derived input function was generated by correcting the aorta signal for plasma–to–whole-blood partitioning and for the percentage of intact ^68^Ga-exendin4 during the scan (based on a population estimate).

The kinetic data were fitted to different compartmental models and graphical analyses, including 1- and 2-tissue-compartment models and Patlak and Logan analysis. On the basis of the fitting, performance, and complexity of the models (Supplemental Tables 1–5), Patlak graphical analysis was selected as the optimal analysis method for the dynamic PET data. Furthermore, ^68^Ga-exendin4 triggers internalization in GLP1R-positive tissues, leading to intracellular ^68^Ga trapping (Supplemental Figs. 1A–1B), theoretically fulfilling the irreversible binding criteria during the time of the scanning as assumed in Patlak analysis. The ^68^Ga-exendin4 net uptake rate (K_i_) was estimated using Patlak graphical analysis in Microsoft Excel (*14*).

K_i_ (mL/[mL⋅h]) was considered a measurement of the concentration of ^68^Ga-exendin4 binding in the pancreas (likely proportional to the GLP1R density in the pancreas). The total GLP1R content was thus estimated by multiplying K_i_ (mL/[mL⋅h]) by the pancreatic volume (mL) as segmented from the PET/CT images.

### Statistics

Data on a group level are reported as mean ± SD. Differences between groups were assessed by 1-way ANOVA. Correlations were assessed by linear regression and the Pearson correlation coefficient (GraphPad Prism for Mac [Apple], version 8.0).

### Data and Resource Availability Statements

The data that support the findings of this study are available from Sanofi, but restrictions apply to the availability of these data, which were used under license for the current study and therefore are not publicly available. Data are, however, available from the authors on reasonable request and with the permission of Sanofi.

## RESULTS

### NHP PET Dose Escalation Study

**FIGURE 1.**
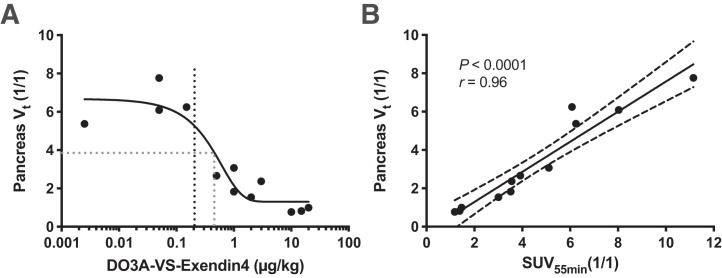
In vivo binding of ^68^Ga-exendin4 in NHP pancreas. (A) Dose escalation studies demonstrated strong binding in pancreas at mass doses below 0.2 µg/kg (black dotted line), which was progressively blocked by coinjection of increasing amounts of unlabeled DO3A-VS-exendin4 precursor peptide. A 50% blocking dose is indicated by gray dotted line. (B) There was strong correlation between V_t_ (obtained from dynamic 90-min scan and requiring blood plasma input signal) and SUV_55 min_, indicating that just static scan from 50 to 60 min can replace dynamic scan.

^68^Ga-exendin4 exhibited visually strong binding and a high V_t_ in the pancreas on all scans coinjecting up to a 0.2 µg/kg dose of unlabeled DO3A-VS-exendin4 precursor peptide (Fig. 1A). There was already a sharp decrease in binding at a peptide mass dose of 1 µg/kg, whereas doses in excess of 10 µg/kg reduced the pancreas binding to the background level. The dose inducing a 50% decrease in binding (the in vivo half-maximal inhibitory concentration) was estimated to be approximately 0.45 µg/kg, indicating very high potency.

Binding was also estimated with a simple semiquantitative SUV measurement of pancreatic binding during the static time frame from 50 to 60 min after injection (SUV_55 min_). SUV_55 min_ correlated strongly with V_t_ (*P* < 0.0001, *r* = 0.96) (Fig. 1B).

### Binding and Biodistribution in Individuals with T2D

**FIGURE 2.**
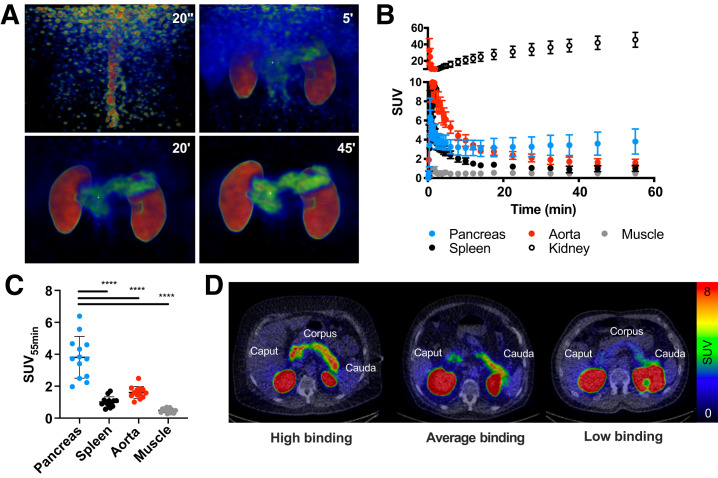
Abdominal biodistribution of ^68^Ga-exendin4 in humans with T2D. (A and B) ^68^Ga-exendin4 was rapidly distributed, followed by washout from most tissues except pancreas and kidneys (average of 13 individuals). Representative maximum-intensity-projection PET images are shown. (C) There was strong variability in pancreas binding, which was not reflected in other tissues. (D) Representative transaxial PET/CT images demonstrate high, average, or low pancreas binding of ^68^Ga-exendin4. ****indicates *P* < 0.0001.

^68^Ga-exendin4 was rapidly distributed in the abdominal tissues after intravenous administration (Figs. 2A and 2B). Initially, the aorta and the left ventricle (i.e., blood signal) were clearly seen, followed by uptake in the pancreas, kidney, and liver in the first 5 min. For the remainder of the scan (60 min), increasing uptake in the pancreas and kidney continued, whereas the remaining tissues demonstrated clearance.

After 60 min, the uptake of ^68^Ga-exendin4 (assessed as SUV_55 min_) was clearly higher in the pancreas than in, for example, the spleen, which has high perfusion comparable to that of the pancreas but is devoid of GLP1R (Fig. 2C). There was marked variability, almost 4-fold, in the magnitude of the pancreas uptake in different individuals (Figs. 2C and 2D). Importantly, this variability was not reflected in other abdominal tissues or the blood signal, indicating it is not due to general differences in biodistribution or metabolism.

^68^Ga-exendin4 uptake was obvious in all 3 pancreatic segments—caput (head), corpus (body), and cauda (tail)—but tended to be higher in the cauda. However, separate delineation of the cauda was difficult because of potential spillover from the kidney in some individuals.

### Metabolic Stability, Self-Blocking, and Protocol Simplification

After administration, ^68^Ga-exendin4 demonstrated high stability in the blood, with more than 90% of the intact tracer being present in plasma at 60 min (Table 2). ^68^Ga-exendin4 also exhibited high plasma partitioning (≈1.8) throughout the scan; that is, most tracer was free in the plasma and available for distribution into tissue (Table 2). The net K_i_ of ^68^Ga-exendin4 in the pancreas was calculated by Patlak graphical analysis, using the metabolite-corrected arterial plasma curve as input (Table 3).

**TABLE 2.** Metabolic Stability and Blood Plasma Ratio of ^68^Ga-Exendin4 in Individuals with T2D (*n* = 3)

Time (min)	Intact peptide (%)	Plasma-to-blood ratio (1/1)
5	98.1 ± 0.9	1.76 ± 0.06
30	95.2 ± 0.4	1.80 ± 0.05
60	90.1 ± 1.4	1.79 ± 0.05

**TABLE 3.** Patlak Graphical Analysis K_i_ Net Uptake Rate and Goodness of Fit for All Individuals Examined with ^68^Ga-Exendin4

Individual	Volume (mL)	SUV_55 min_ (1/1)	K_i_ (mL/[mL⋅h])	*R* ^2^
1	78	3.4	0.60	0.99
2	69	6.4	0.93	1.0
3	48	2.0	0.38	0.97
4	66	3.8	0.62	0.98
5	63	3.7	0.63	0.98
6	78	4.3	0.83	0.99
7	59	2.2	0.45	0.99
8	73	3.7	0.59	1.0
9	57	2.5	0.38	0.99
10	49	2.6	0.43	0.97
11	75	4.6	0.83	0.99
12	135	4.7	0.58	0.99
13	117	5.6	0.97	1.0

SUV_55 min_ and pancreas volumes in same individuals are included for comparison.

**FIGURE 3.**
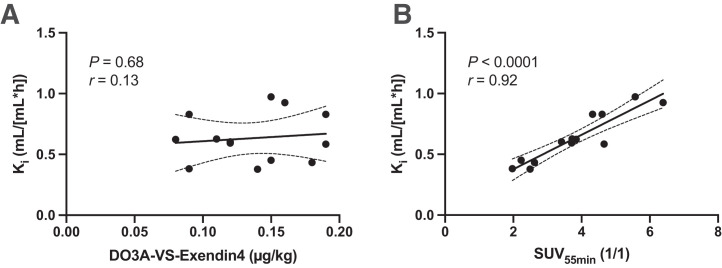
In vivo binding of ^68^Ga-exendin4 in human T2D pancreas. (A) There was no obvious self-blocking mass effect at peptide doses below 0.2 μg/kg (i.e., no negative correlation). (B) In human pancreas, there was strong correlation between model parameter obtained from dynamic scan including blood (Patlak K_i_) and SUV_55 min_, indicating that static scan is sufficient for accurate quantification.

As per the study design, all individuals were administered less than a 0.2 µg/kg dose of DO3A-VS-exendin4 peptide to avoid self-blocking due to the high potency of the compound for GLP1R. As predicted, there was no negative correlation between the peptide mass dose and the net K_i_, indicating that the self-blocking at these levels was also negligible in humans (Fig. 3A).

SUV_55 min_ correlated well with the net K_i_ (*P* < 0.0001, *r* = 0.92), indicating that the lengthy dynamic scanning and invasive blood sampling may be replaced by shorter static scanning from 50 to 60 min after tracer administration with only a minor loss in accuracy (Fig. 3B).

### Correlation of Binding with Age and BMI

**FIGURE 4.**
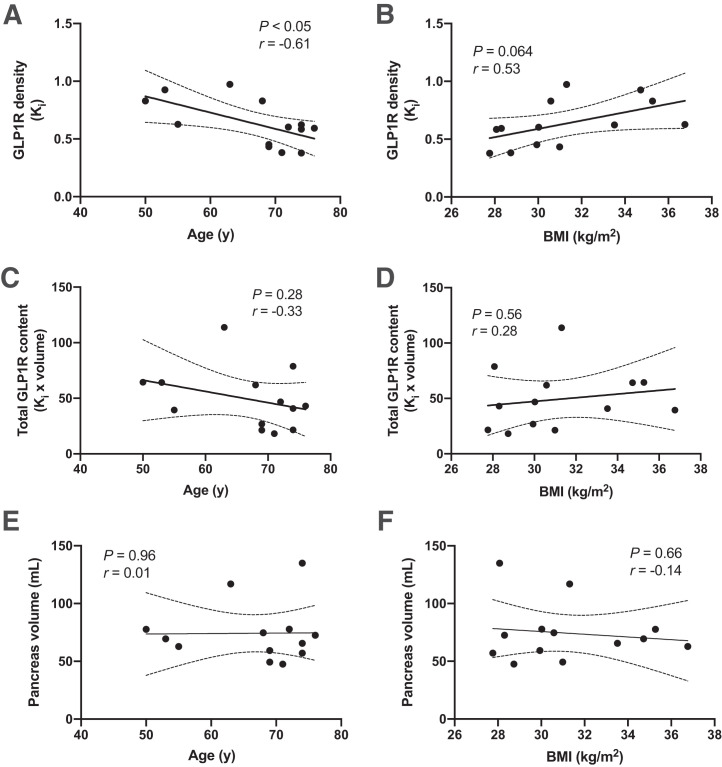
Correlation of ^68^Ga-exendin4 pancreas binding with biometric parameters. (A and B) Pancreatic GLP1R density (concentration of ^68^Ga-exendin4 binding) correlated negatively with age of examined individuals (A) and exhibited tendency to correlate with BMI (*P* = 0.064) (B). (C and D) However, total pancreas GLP1R content (i.e., ^68^Ga-exendin4 concentration multiplied by volume) did not correlate with age (C) or BMI (D) of participants. (E and F) Similarly, size of pancreas did not correlate with either age (E) or BMI (F) in this study.

There was a distinct variability in the ^68^Ga-exendin4 net K_i_ in the pancreas. We therefore explored the potential correlation between ^68^Ga-exendin4 binding and the characteristics and biometrics of patients. The variability in ^68^Ga-exendin4 binding as assessed by K_i_ (i.e., GLP1R density) correlated negatively with the age of the participant (*P* < 0.05, *r* = −0.61) (Fig. 4A). The same correlation with age, but using SUV_55 min_, was −0.50 (*P* = 0.082). Furthermore, there was a tendency toward a positive correlation between ^68^Ga-exendin4 binding in the pancreas and the BMI of the participants (*P* = 0.064, r = 0.53) (Fig. 4B). Similarly, the correlation between BMI and SUV_55 min_ was 0.43 (*P* = 0.145). However, when we estimated the total ^68^Ga-exendin4 binding (i.e., total GLP1R content) by multiplying by the pancreatic volume, no correlations remained (Figs. 4C and 4D). The pancreatic volume of the participants ranged between 48 and 135 mL and did not correlate with, for example, age or BMI in this cohort (Figs. 4E and 4F).

## DISCUSSION

In this study, we demonstrated the binding and biodistribution of ^68^Ga-exendin4 in the pancreas of humans with T2D. Furthermore, we developed an optimized and simplified ^68^Ga-exendin4 scanning protocol based on these initial experiences.

Native exendin4 is a highly potent GLP1R agonist, with subnanomolar potency (*18*). Radiolabeled ^68^Ga-exendin4 has excellent specificity for GLP1R (Supplemental Fig. 1A) and affinity in the nanomolar range (*19*). In the absence of suitable antagonists, agonists such as exendin4 can be used as PET tracers, but care must be taken to avoid unwanted pharmacologic effects. We have previously demonstrated that ^68^Ga-exendin4 also exhibits self-blocking at low administered precursor peptide mass doses in NHPs. Doses of around 0.5 µg/kg elicit up to 50% blocking of the signal. This result is not entirely unexpected, given the high potency of ^68^Ga-exendin4 for GLP1R. In fact, peptide mass effects of similar magnitude were seen for 2 other gut hormone peptide PET tracers, ^68^Ga-DO3A-VS-Tuna-2 (a glucagon receptor agonist) and ^68^Ga-S02-GIP-T4 (a glucose-dependent insulinotropic polypeptide receptor agonist), both with a potency in the picomolar range (*20*,*21*).

Here, we further demonstrated from NHP data that a peptide mass effect can be avoided at less than 0.2 µg/kg doses of coinjected DO3A-VS-exendin4. This finding influenced the design of the clinical study, which subsequently showed minimal mass effects. Thus, any future clinical studies with ^68^Ga-exendin4 should aim to administer no more than a 0.2 µg/kg dose of precursor peptide. This limitation will likely entail an optimized automated radiochemistry setup (*16*), since a molar activity of at least 20–30 MBq/nmol will be required. Furthermore, even at an optimal molar activity, 0.2 µg/kg will probably correspond to no more than 50–100 MBq to be administered; this, in turn, necessitates highly sensitive PET instrumentation for sufficient image quality.

Quantitative PET assessment usually entails quite complex correction of imaging data, including dynamic imaging acquisition of a long duration, arterial sampling, and metabolic stability measurement. Such complicated scanning protocols present logistical challenges and are uncomfortable for the patient. Additionally, the analysis is time-consuming and requires specialized software and expertise. Semiquantitative measurements derived from static scanning protocols, such as SUVs, on the other hand, are simpler from the point of view of both the patient and the hospital, in addition to allowing for rapid analysis.

In both the NHP and the clinical studies, we could show that the static SUV (SUV_55˜min_) exhibited a very strong correlation with the modeled parameter (which was derived from the full dynamic scanning protocol including blood sampling and metabolic stability correction of the input signal). Thus, it can be assumed that a shortened static scan at 50–60 min after injection provides reasonable accuracy in the assessment of GLP1R density in the pancreas, with only a minor loss in reproducibility compared with a full dynamic scan. In many cases, this trade-off may be acceptable to substantially increase patient comfort and simplify scanning procedures and logistics. However, ^68^Ga-exendin4 uptake as assessed by SUV_55 min_ did not correlate as well with, for example, age as did Patlak net K_i_, indicating that the simplification of the analysis and scanning protocol may reduce the power of the assessment.

A limitation of this study is the lack of a test–retest assessment, that is, repeated scanning of the same subject without any intervention in between. Such data would provide important information on the reproducibility of the assessment—either analyses by SUV_55 min_ or analyses by Patlak net K_i_. Unfortunately, test–retest scanning was not possible because of the design of the full occupancy study (clinicaltrials.gov identifier NCT03350191) (*12*).

In short, future PET studies using ^68^Ga-exendin4 for assessment of GLP1R in the pancreas should aim to coinject less than a 0.2 µg/kg dose of peptide. Moreover, patient comfort and scanning throughput could be increased by limiting the scanning protocol to a static scan approximately 60 min after injection.

Despite the rigorous standardization of our scanning protocol, ^68^Ga-exendin4 exhibited a marked variability in pancreatic binding. We further explored potential reasons for this variability. There was a negative correlation with age in this study, indicating a lower GLP1R density in the pancreas of older individuals. A progressive decrease in GLP1R in the mouse brain has previously been reported (*22*). One of the major sources of ^68^Ga-exendin4 binding in the human pancreas is the β cells, which exhibit strong GLP1R expression (*23*). The known decline of β-cell number and function with age (*24*) thus presents a possible mechanism for this reduction in ^68^Ga-exendin4 with increasing age. However, when we calculated the total GLP1R content (by multiplying ^68^Ga-exendin4 binding by the pancreas volume), the negative correlation did not prevail.

We also visually observed a general trend toward higher ^68^Ga-exendin4 uptake in the cauda of the pancreas in many individuals. This is again interesting in the context of β cells, since the cauda has been shown to exhibit approximately 2-fold the islet density of the caput and the corpus (*25*). Thus, we observed several features of the ^68^Ga-exendin4 binding that were consistent with known β-cell distribution and function. Additionally, the large variability in ^68^Ga-exendin4 observed here (almost 4-fold) is in line with the large variability in β-cell mass seen in individuals with T2D as assessed by biopsy morphometric studies (*26*). More studies dedicated to this question are required to further establish the association between ^68^Ga-exendin4 binding and the β-cell mass in the human pancreas in T2D.

We observed a trend toward a positive correlation between ^68^Ga-exendin4 binding and BMI in this cohort; that is, an increasing pancreatic GLP1R density correlated with a higher BMI. It is difficult to assess whether this is a reasonable observation, as there are limited data on GLP1R density in the pancreas of obese subjects—likely because of the challenge of obtaining diverse biopsy material of high quality, lack of reliable monoclonal antibodies for GLP1R, and the time-consuming effort required for sectioning, staining, and analyzing a sufficiently large dataset to draw firm conclusions. Again, the correlation with BMI did not remain when the total GLP1R content in the pancreas was estimated by multiplying by volume.

A limitation of the study and the correlations discussed above is the imbalanced sex distribution of the study population. Of the 13 included individuals, 12 were male and only 1 was female. Pancreatic binding in the female individual in this study was around the population average. However, the correlations seen here may reflect mainly the male population.

^68^Ga-exendin4 could conceivably serve as a biomarker for response to therapy. GLP1 efficacy studies sometimes delineate subgroups of nonresponding individuals—both in studies targeting populations with diabetes (*27*) and in studies targeting populations with neurologic disease (*28*). In this small cohort, some of the participants (*n* = 7) completed 3 wk of up-titration with the dual GLP1/glucagon agonist SAR425899, and all responded to treatment with blood glucose lowering and weight reduction, regardless of exhibiting a wide range of GLP1R density in the pancreas (*12*). Therefore, excessively up- or down-regulated GLP1R in the pancreas does not seem to predict GLP1 agonist responders or nonresponders in this admittedly small cohort.

## CONCLUSION

We present an optimized and simplified ^68^Ga-exendin4 scanning protocol to enable reproducible imaging of GLP1R in the human pancreas. ^68^Ga-exendin4 PET may enable longitudinal quantification of changes in pancreatic GLP1R during the development of T2D, as well as target engagement studies of novel GLP1 agonists.

## DISCLOSURE

The clinical study was sponsored by Sanofi and was performed in collaboration with Antaros Medical AB. The NHP study was sponsored by JDRF, Diabetesfonden, and Barndiabetesfonden. Olof Eriksson’s position is funded by Science for Life Laboratory and the Swedish Research Council (2020-02312). Torsten Haack, Martin Bossart, Joachim Tillner, and Michael Wagner are employees of Sanofi-Aventis and may hold shares or stock options in the company. Olof Eriksson, Iina Laitinen, Stefan Pierrou, and Lars Johansson are employees of Antaros Medical AB. Philip J. Larsen is an employee of Bayer Pharmaceuticals. Jan Erik Berglund is an employee of CTC AB. No other potential conflict of interest relevant to this article was reported.

## Notes

KEY POINTS **QUESTION:** Can GLP1R density in the pancreas of individuals with T2D be evaluated by ^68^Ga-exendin4 PET? **PERTINENT FINDINGS:** ^68^Ga-exendin4 binding in the T2D pancreas was high, consistent with the known expression patterns of GlP1R in the abdominal area. GLP1R density correlated inversely with the age of the individual and tended to correlate positively with BMI. **IMPLICATIONS FOR PATIENT CARE:** ^68^Ga-exendin4 PET may enable quantification of longitudinal changes in pancreatic GLP1R during the development of T2D, as well as target occupancy in drug development studies.
